# Occupational Safety Climate in the Swedish Equine Sector

**DOI:** 10.3390/ani12040438

**Published:** 2022-02-11

**Authors:** Cecilia Lindahl, Åsa Bergman Bruhn, Ing-Marie Andersson

**Affiliations:** 1 Department of Agriculture and Food, RISE Research Institutes of Sweden, P.O. Box 7033, SE-75007 Uppsala, Sweden; 2 Work Sciences, Dalarna University, SE-79188 Falun, Sweden; ima@du.se

**Keywords:** equestrian sport, horse industry, NOSACQ-50, riding school, safety climate assessment, safety culture, trotting, work environment

## Abstract

**Simple Summary:**

Handling and working with horses is a hazardous activity, as horses are large, powerful and unpredictable animals, and the equine sector is reported to have a relatively high occupational injury rate. The safety climate in a workplace is a good indicator of safety performance because it captures employees’ attitudes and perceptions of safety at a specific point in time. The safety climate in the equine sector is a relatively unexplored area, though research could be a fruitful approach to improving occupational safety. In this study, the safety climates at six Swedish riding schools and six trotting stables were assessed using a questionnaire on the safety climates at these workplaces and complementary interviews. The riding schools and trotting stables all had favourable safety climates and employees were well-aware of the injury risks in their work environment. The main aspect of the workplace safety climate identified as needing improvement was the workers’ prioritisation and non-acceptance of risk, and their propensity to take calculated risks. Minor injuries were considered part of the job, indicating a need to communicate the importance of such injuries. This aspect of the equine safety climate should be targeted to improve future safety. The management should promote a culture of safety awareness at all levels of the organisation, making safety an integral part of daily work. Accident investigations should be performed systematically to learn from negative events, identify factors contributing to injuries and develop strategies for injury prevention.

**Abstract:**

The Swedish equine sector is considered a high-risk work environment, with relatively high injury rates and high severity of injuries. General safety research has identified a correlation between the safety performance and safety culture, but little is known about the intricacies of the safety culture in the Swedish equine sector, especially concerning managers’ and employees’ perceptions of their work environment. The safety climate assessment is recognised as an effective tool for identifying potential problems in the workplace, thus enhancing safety behaviour and decreasing the frequency and severity of injuries. The aim was to evaluate the safety climate at riding schools and trotting stables through the Nordic Safety Climate Questionnaire (NOSACQ-50) diagnostic tool, and to get a better understanding of the workers’ perceptions regarding safety and safety management at their workplace through complementary interviews. The results showed that the safety climate was generally positive and that employees were aware of the risks relating to their work. Riding schools commonly had routines in place for risk assessment and work environment management, but such routines were often lacking at trotting stables, indicating inadequate prioritisation of safety by the management. The main area that should be targeted to improve safety in the sector is employees’ prioritisation and non-acceptance of risks. Proactive instead of reactive safety management should be promoted, where safety is an integral part of daily work and all employees are encouraged to identify factors contributing to occupational injuries and develop strategies for injury prevention.

## 1. Introduction

In Sweden and many other European countries, the equine sector has grown strongly in recent years. According to statistics from the Swedish Board of Agriculture [1], the number of horses in Sweden increased by 8–17% between the years 2004 and 2016. At present, there are approximately 355,500 horses in Sweden, of which 18,300 are owned by riding schools, making it one of the countries with the highest number of horses per capita (36 per 1000 inhabitants) in Europe [1]. The Swedish equine sector is diverse and encompasses a wide variety of activities, including businesses related to breeding, competition, tourism and training, and not-for-profit activities such as association-run riding schools and leisure. The annual turnover of the Swedish horse sector is SEK 31 billion, and it provides approximately 17,000 full-time jobs [2].

There are about 860 Swedish riding clubs, more than half of which also run a riding school [3], and just over 3700 individuals have some form of trotting licence, of whom 400 have an A-licence, meaning that they are professional trotting horse trainers [4]. Horse stables and riding schools have a skewed gender distribution [5], with 90% of the workforce being women, and many are also young. Professional trotting trainers are predominantly men, while the majority of trotting horse grooms are women.

Work environment issues are a major concern for the equine sector, which is labour-intensive. The sector is generally highly traditional, with many work tasks still performed manually with old-fashioned tools and equipment like wheelbarrows, manure forks and brooms [6,7,8]. Productivity and profitability have historically not been a priority, leading to low interest and a lack of willingness to invest in mechanisation. Thus, many work tasks are still performed manually, leading to high workloads and physical strain. Furthermore, handling and working with horses is a hazardous activity as horses are large, powerful and unpredictable animals [9,10]. The Swedish equine sector has a relatively high occupational injury rate, but there is a lack of reliable statistics on these occupational injuries. Among animal-related occupational injuries in Sweden, horses are most often cited as the source of the injury [11]. Horse-related injuries are often severe, and according to a review of occupational injury statistics in Sweden, businesses offering ‘riding training and rental of horses’ have the highest injury severity rate among women (i.e. number of sick days per employee and year) of all industries (severity rate of 1.1 compared with a mean of 0.2 for all industries) [12]. The Swedish Work Environment Authority reported one fatal injury and 770 occupational injuries involving horses during 2009–2013, of which 332 injuries resulted in sick leave of more than 14 days [13]. During 2016–2020, a total of 301 occupational injuries requiring sick leave were reported by riding and trotting schools alone, according to the Swedish Work Environment Authority’s injury statistics [14]. The official statistics on occupational injuries are most likely just the tip of the iceberg as there is a high level of under-reporting, especially of less severe injuries. Heinrich’s triangle theory [15], as refined by Bird [16], postulates that there is a numerical relationship between near misses (i.e., events that could have resulted in an injury in different circumstances) and minor and major injuries, with the incidence of near-misses and minor injuries being significantly higher than that of major and fatal injuries. Several studies worldwide have identified the equine sector as a high-risk work environment [17,18,19,20,21]. Despite this, research focusing on the work environment and safety in the equine sector is limited.

In Europe, the European Framework Directive on Safety and Health at Work [22] is seen as the core for creating safe workplaces. As part of this, regulations on systematic work environment management in Sweden were introduced in 2001, to ensure employee rights to health and safety and stimulate safe working conditions [23]. The regulations require ongoing efforts to introduce safety improvements to the workplace and allow employees to contribute to these improvements. The requirements include examining and assessing risks, planning measures to counter identified hazards and investigating ill health, injuries and incidents [23]. Due to the high injury prevalence in the equine sector, during 2016–2017, the Swedish Work Environment Authority conducted a targeted supervisory effort focusing on the work environment management in the sector. In total, deficiencies were identified in 49% of the inspections carried out within that project, and it was concluded that the equine sector’s view of the work environment must fundamentally change to reduce ill-health in the sector [13]. Since then, initiatives such as training and education in work environment management have been introduced by some equine organisations, but to achieve broader and more long-lasting improvements in workplace safety, the entire sector needs to be involved. 

The safety culture has been identified as a key factor determining the importance of workplace safety within an organisation. The safety culture can be defined as “the product of values, attitudes, competencies and behavioural patterns at individual and group level, which determine commitment to, and style and competence of, an organisation’s health and safety program” [24]. A safety culture is built and sustained over time. A term closely related to the safety culture is the safety climate, which is a snapshot of the safety culture of an organisation at one specific point in time. Kines et al. [25] defined the safety climate as “workgroup members’ shared perceptions of management and workgroup safety-related policies, procedures and practices”. The safety climate can change quickly, e.g. due to an incident or new safety procedures in the workplace, and is, therefore, a good indicator of the safety performance at a specific time. Several studies have shown that a good safety climate and a strong safety culture lead to fewer occupational injuries and have a positive effect on safety in the workplace [26,27]. The long-term outcomes can also include increased productivity and lower costs [28].

According to Schein [29], one of the most important tasks for leaders of organisations is to be a carrier of culture and thus influence how employees in the organisation think and act. Research indicates that good leadership may be imperative for managing occupational risks [30], and thus the leadership and culture should be studied together as they are so closely linked [31,32]. Löfving et al. [33] claimed that this is particularly evident in small- and medium-sized organisations, where the relationship between the leadership and culture is even more evident, resulting in difficulty in recognising where the organisational culture ends and leadership begins. 

Research on the safety climate has been increasing during the 21st century [34] and has produced growing evidence that the safety climate is associated with safety practices [35], accidents [36,37], musculoskeletal disorders [38] and unsafe behaviour [39,40,41,42]. Therefore, assessing the safety climate can be an effective tool for evaluating the safety performance in a workplace, identifying safety problems and planning measures to improve safety. A safety climate assessment also can provide proactive indicators of safety problems before they cause injuries, in contrast to traditional retroactive safety measures such as accident and near-accident reports. 

The safety culture and climate in the equine sector is an unexplored research area, despite the high-risk work environment in the sector. A recent survey involving respondents from 25 different countries found a general acceptance of injury risks during horse interactions, and some respondents even de-emphasized the importance of safety-first principles [18]. The results also showed that respondents who derived an income from horse-related activities had a higher propensity for risk-taking in general, and risk-taking in sports and occupational settings in particular. In light of these results, increasing the focus on the safety culture within horse-related occupations could represent a fruitful approach to improving occupational safety and health in the equine sector.

There is a general lack of research related to the equine sector, particularly in terms of issues concerning the working environment, health, safety and working conditions in horse-related professions. Understanding the safety climate in the equine sector is an essential first step to devising approaches to enhance safety, and in the long-term, increase the sustainability of horse-related professions. This study aimed to evaluate the safety climate at riding schools and trotting stables, to get a better understanding of employees’ perceptions regarding safety and safety management at their workplace. Riding schools and trotting stables were chosen as they represent an educational and performance context, respectively. The study also included the employer perspective, to compare the perceptions of work environment management held by managers and employees.

## 2. Materials and Methods

An explanatory sequential mixed-methods design [43] was used in the study. First, quantitative data were collected and analysed to gain an understanding of the safety climate in the equine sector. For explanation and a deeper understanding of the results, additional quantitative and qualitative data were then collected and analysed. Finally, the results were summarized and interpreted. A summary of the data collection procedure and sample size is presented in Table 1. 

Riding schools and trotting stables were selected for analysis to cover safety aspects relating to, respectively, horse riding as a training experience and equine competition and performance. The riding schools and trotting stables were chosen through a combination of purposive and convenience sampling, based on the geographical location, general engagement in work environment management at the workplace and willingness to participate in the study. The aim was to include workplaces of different sizes, staff sizes and representing different gender and age groups. A reference group comprising representatives from e.g., the Swedish Horse Industry Foundation (HNS) and the Swedish Equestrian Federation, with good knowledge of the Swedish equine sector, aided in selecting suitable workplaces. The managers of the workplaces were contacted by phone, informed about the project and invited to participate. If they agreed, visits to the workplaces were planned. All permanent employees at each workplace were invited to complete the Nordic Safety Climate Questionnaire (NOSACQ-50) and participate in interviews.

### 2.1. Study Participants

Data were collected from the employees and managers of 12 equine businesses (six riding schools and six trotting stables) located in Central Sweden. The riding schools had 4–15 employees, while the trotting stables had 3–8 employees. The employees worked full-time or different degrees of part-time. Demographic characteristics of the questionnaire respondents and interviewees are summarised in Table 2. 

### 2.2. Employee Questionnaire

All permanent employees at the 12 selected workplaces were asked to complete the Nordic Safety Climate Questionnaire (NOSACQ-50) regarding the safety climate at their workplace. NOSACQ-50, which was developed by a Nordic network of occupational safety researchers (for development and validation, see Kines et al. [25]), is a useful tool for assessing the safety climate at workplaces and can be used for comparative studies to evaluate the safety climate status and safety climate interventions. The NOSACQ-50 has been used in multiple studies across sectors, countries and applications, and has been confirmed as a reliable approach for diagnosing the occupational safety climate [26,27,44,45]. Results from studies around the world are collected in an international database to allow for benchmarking and further development. Data from the NOSACQ-50 database are presented in Section 3.1.

The questionnaire consists of 50 statements describing seven safety climate dimensions, i.e., a group’s shared perceptions, as presented in Table 3.

The NOSACQ-50 statements can be divided into two types: positively formulated statements and reversed statements. For each statement, the respondents were asked to rate their level of agreement according to a Likert scale with four terms: strongly agree, agree, disagree and strongly disagree. For positive statements, the terms corresponded to a rating scale of 1 (strongly disagree) to 4 (strongly agree), while the rating scale was the opposite for the reversed statements. A mean score for each dimension was calculated from the statements. To interpret the questionnaire results, each dimension was evaluated according to the criteria suggested by the National Research Centre for the Working Environment of Denmark (on the NOSACQ-50 website) (Table 4) [46].

In addition to the NOSACQ-50 statements, further background questions were included regarding the respondent’s birth year, gender, work experience and whether they held a managerial role at the workplace.

### 2.3. Manager Questionnaire

The manager of each workplace was asked to complete a systematic work environment management (SWEM) questionnaire regarding the management of their workplace. A total of 11 managers responded to the questionnaire (Table 2). The SWEM questionnaire, which was developed by the Swedish Work Environment Authority, consists of 12 statements based on the Swedish provisions and general recommendations for systematic work environment management [23]. Two of the statements were selected for use in this study as they relate directly to safety management, while the other 10 statements were considered not relevant to the context of the study. The selected statements were:

- At our workplace, we regularly investigate the working conditions and assess the risks of any person being affected by ill-health or accidents at work. The risk assessment is documented in writing.
- At our workplace, the employer investigates the causes in the event of an employee suffering ill-health or an accident at work, or in the event of a serious incident at work, so that risks of ill-health and accidents can be prevented in the future.

For each statement, the respondents were asked to rate their level of agreement according to a six-point Likert scale ranging from 1 (strongly disagree) to 6 (strongly agree).

### 2.4. Interviews with Employees 

Qualitative data were collected between February 2019 and May 2021 (2–12 weeks after the quantitative data collection), through 47 individual interviews with employees at the six riding schools and six trotting stables (Table 2). Semi-structured interviews were conducted using an interview guide covering the topics: (1) how risk is managed in the workplace, (2) perceptions of risk and safety factors at work and (3) prevention strategies. The interviews were used to explore employees’ perceptions of the safety climate, with a focus on the consequences for sustainable working life. The interviewees were encouraged to “tell their story”, starting from their first interest and activity related to horses and the equine sector and continuing to their current situation, as well as offer their ideas and thoughts about the future, to gain an understanding of their experiences. Thus, the interviews covered the work environment in a wider perspective that is not presented fully here as it was beyond the scope of the present analysis. Only responses relating directly to accidents and incidents, safety, risk perception and awareness, safety management and risk assessment were included in the analysis.

The interviews lasted from 11 to 73 minutes (including all topics), with an average duration of 36 minutes. The interviews were performed with visual contact, either in a physical meeting at the workplace (27 interviews) or digitally using the software Microsoft Teams, with cameras activated, due to the coronavirus pandemic (20 interviews). Two researchers were present during interviews, with one responsible for guiding the interview and one responsible for taking notes. All interviews except for one were digitally recorded, and they were transcribed afterward from the recordings.

All interviewees were offered the opportunity to read the transcript of their interview to allow them to verify the content, clarify their intentions, correct errors and provide additional information. The majority of the interviewees took this opportunity, and two made additions to the content. Analysis of the qualitative data was conducted by the researchers independently and the results were then discussed in-depth, to ensure congruence between the interview data and results, and thus avoid bias. 

### 2.5. Data Analysis

Data obtained by the NOSACQ-50 questionnaire were analysed by calculating the mean value for each dimension and participant according to the method described on the NOSACQ-50 website [47]. Each respondent’s mean value per dimension then formed the basis for calculating the mean value for dimensions Dim1–Dim7 for the entire workplace. A t-test was used to analyse differences in the safety dimension scores between riding schools and trotting stables, females and males, age groups (20–35 years old and ≥36 years old) and work experience (0–5 years and ≥6 years). All statistical analyses were performed in the software package SPSS Statistics for Windows, Version 27.0 (IBM Corp., Armonk, NY, USA). Qualitative data were used for further analysis of the NOSACQ-50 dimensions. The SWEM questionnaire results were re-calculated as a mean value for riding schools and trotting stables, respectively, and the outcomes were mainly used as an explanation for Dim7 by adding the manager’s self-assessment of SWEM at the workplace. These data were considered too limited for statistical analysis to be meaningful.

The qualitative content analysis [48], based on deductive reasoning, was inspired by systematic text condensation according to Malterud [49] and thematic analysis according to the recommendations of Braun and Clarke [50]. Initially, the entire interview transcripts were read separately and repeatedly, to get an overview of the content. In the next step, smaller text units with similar content were identified and coded, and the codes were collated into themes. Six themes were identified, of which five fell into specific safety climate dimensions and thus contributed to a deeper understanding and explanation of the NOSACQ-50 results. One theme (horsemanship) was not specifically related to any dimension in NOSACQ-50, and so it is presented as a separate theme in the results section.

## 3. Results

### 3.1. NOSACQ-50 Results

The mean scores obtained for the seven safety dimensions in NOSACQ-50 are presented in Table 5. In total, all except one safety dimension had a level interpreted as good. The exception, safety dimension Dim5 (workers’ safety prioritisation and risk non-acceptance), had the lowest score, indicating a need for improvement.

Comparing the results for riding schools and trotting stables separately revealed that riding schools had a good level for all safety dimensions except Dim5, which needed certain improvement, while trotting stables had one dimension (Dim5) with a level interpreted as fairly low and in need of improvement, and two further dimensions, Dim1 (management safety prioritisation, commitment and competence) and Dim7 (trust in the efficacy of safety systems), requiring certain improvement (Table 5).

The t-test results for differences in the safety dimension scores between riding schools and trotting stables, females and males, age groups (20–35 years and ≥36 years old) and work experience (0–5 years and ≥6 years) are presented in Table 6. Significant differences between riding schools and trotting stables were found in the safety dimensions Dim5 (workers’ safety prioritisation and risk non-acceptance), Dim6 (safety communication, learning and trust in co-workers’ safety competence) and Dim7 (trust in the efficacy of safety systems), where riding schools scored higher for all three dimensions. No differences were found between females and males or age groups 20–35 years and ≥36 years. When comparing the safety dimension scores between respondents with work experience of 0–5 years and work experience of six years or more, workers with less experience had a significantly higher score in safety dimension Dim4 (workers’ safety commitment). However, the mean scores were above 3.3 for both groups, meaning that the safety dimension level was interpreted as good.

**Figure 1:**
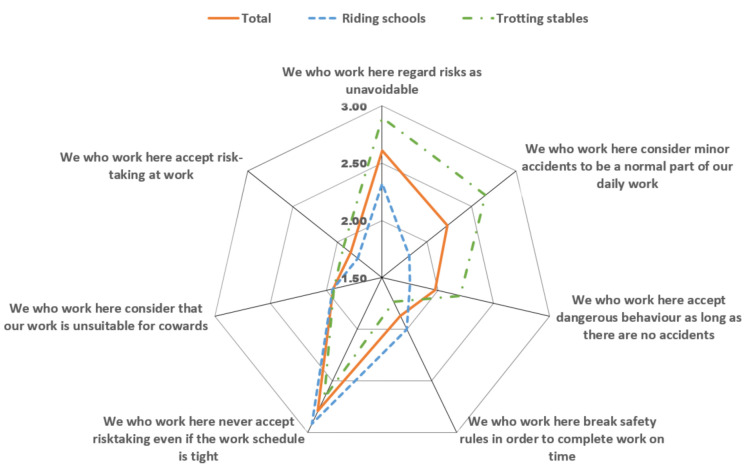
Scores (mean values) given for each of the seven statements within dimension 5 in the Nordic Safety Climate Questionnaire (NOSACQ-50) by all respondents and by workers at riding schools and trotting stables as separate groups. Score range from 1 (strongly disagree) to 4 (strongly agree).

Dimension Dim5 had the lowest score for riding schools and trotting stables and there was a significant difference between these groups. Therefore, the seven statements included in Dim5 were studied in greater depth. The mean scores for each of the seven statements are presented in Figure 1. As can be seen from the diagram, the difference between riding schools and trotting stables was particularly evident for three statements: “We who work here regard risks as unavoidable”, “We who work here consider minor accidents to be a normal part of our daily work” and “We who work here accept dangerous behaviour as long as there are no accidents”. All three statements were given a higher score, indicating stronger agreement with the statements, by workers at trotting stables than by workers at riding schools (Figure 1).

### 3.2. SWEM Questionnaire Results 

The participating managers were asked to answer two SWEM questions regarding systematic work environment management at their workplace.

For the first statement, about regularly investigating working conditions and assessing the risks of ill-health or accidents at work, managers at riding schools (*N* = 6) had a mean value of 5.2 (range 3–6), indicating relatively strong agreement with the statement. Managers at trotting stables (*N* = 5) agreed to a lesser extent with the first statement, with a mean value of 2.4 (range 1–4).

For the second statement, about having routines regarding the investigation of causes in the event of an employee suffering ill-health or an accident at work, or in the event of a serious incident at work, managers at riding schools again showed stronger agreement (mean value 5.3, range 4–6) than managers at trotting stables (mean value 4.2, range 2–4), but the difference was lower than for the first statement.

### 3.3. Interviews with Employees

Six themes were identified in the qualitative analysis of interview responses. These were: management, risk awareness/acceptance, knowledge/experience, communication/information, safety routines and horsemanship. Data relating to the first five themes were sorted under their related dimension (Table 7), while horsemanship was considered not to be directly related to any of the dimensions and was, therefore, assessed separately.

#### 3.3.1. Management Safety Prioritisation, Commitment and Competence (Dim1)

In interviews, the employees at riding schools generally talked more about their safety prioritisation and that of their colleagues than that of the management. Some of the employees interviewed at riding schools said that formal risk assessments were not conducted in their workplace, but that the management reacted when incidents or accidents occurred and that necessary changes of routines were implemented to increase safety. Thus, safety management was generally described as reactive rather than proactive:

“The safety level is not high, I would say. I think not. Until now… Perhaps you heard about the accident that occurred? Up to that, there was no safety at all […] Now, we have some official forms and stuff, which we haven’t had before. They should have been there a long time ago. It’s surprising they weren’t, I think.” (26 years, male, riding school)

However, there was great variation between the riding schools, with some having work environment and safety policies, regular risk assessments and clear guidelines on safety, indicating prioritisation of safety by management, while others were reported to lack such preventive safety management. One interviewee reported having created his own routines for the work environment and safety as a response to the lack of formalised routines in the workplace.

Employees at trotting stables mentioned the leadership more specifically, with some managers being described as good, knowledgeable and enforcing clear guidelines, while others were described as vague and inexperienced. Employees from two different workplaces said that their manager never talked about safety with the staff. Managerial factors that were mentioned as compromising safety at trotting stables were poor scheduling with too tight a schedule, lack of personal protective equipment provided by the employer and working alone.

#### 3.3.2. Worker’s Safety Prioritisation and Risk Non-Acceptance (Dim5)

Many of the employees we interviewed were aware of the risks in the workplace and acknowledged that handling and working with horses are hazardous activities. Horses were described as friendly but large, and the fact that they are flighty animals that can react unpredictably to any situation was mentioned as a source of injury risk. Getting to know the horses, matching the horse to the handler and following good routines were mentioned as ways of decreasing the risks. Knowledge, experience and developing a sense for the horse were also believed to increase safety (see Section 3.3.5). This is illustrated by the following two responses:

“The great difference between occupations within the horse sector and other physically heavy and challenging occupations is handling the horses.” (28 years, female, riding school).

“Of course, there are risks, but I don’t think about those. But you probably should. They [horses] are animals so anything can happen.” (42 years, female, trotting stable).

The nature of horses was noted to make it difficult to prevent all risks. There was a general acceptance among workers at both riding schools and trotting stables that certain risks are involved when working close to horses. Commonly, the employees interviewed also stated that horse safety and health were prioritised over human safety and health. Minor injuries were expected, for example, being stepped on or run over by a horse was considered inevitable when working with horses. The risk acceptance and normalisation of injuries among interviewees are illustrated by the following statement:

“I’ve been lucky and haven’t had any severe accidents, but I have been kicked and bitten and crushed, and I’ve had my feet crunched. I have fractures in both little fingers, but I haven’t experienced anything serious.” (26 years, female, trotting stable)

Employees at riding schools, in particular, reported strong safety awareness among themselves and their colleagues, and they described how they try to stay one step ahead and foresee when something is about to happen. To “lead by example” and “live as we learn” were other expressions used by workers at riding schools when reflecting on safety in the workplace and their responsibility to teach their pupils to have a safety mindset.

Employees at both riding schools and trotting stables reported that safety was sometimes compromised, often due to a lack of time. One example mentioned was leading horses to the pasture two at a time despite awareness that this greatly increases the risks. This was explained as “a calculated risk” and “conscious risk-taking”. Time pressure was also perceived to increase the risk of making mistakes and being careless, e.g., not checking the horse’s tack or not using personal protective equipment like a helmet, gloves and body protector. One interviewee reasoned thus regarding accidents:

“Most incidents happen due to carelessness. You know what you should do, but you do things another way and then something happens. […] When something happens, you have messed up. It is the human factor, and nothing can be done about it, I think.” (27 years, female, trotting stable).

#### 3.3.3. Safety Communication, Learning and Trust in Co-Workers’ Safety Competence (Dim6)

The employees interviewed generally expressed trust in their co-workers’ safety competence. In particular, several employees at riding schools reported striving to work systematically to overcome work environment issues and said that they are solution-oriented and help each other. Thus, safety was perceived as a shared responsibility and participation was considered important. This was not as clearly stated by the employees interviewed at trotting stables.

Many employees believed that safety awareness is gained through experience and knowledge. Learning from one’s own mistakes or those of others was commonly mentioned as a way to become more aware. Thus, knowledge of how to work safely around horses appears to take time to develop and also involves learning from accidents and incidents. This was a central component of the interviewees’ reasoning regarding horsemanship (see Section 3.3.5). An employee at a riding school said:

“While you gain knowledge from education, it is through experiencing daily work in the business that you really learn the profession.” (28 years, female, riding school).

Another worker with 33 years of experience as a riding instructor explained how experience helps to foresee accidents during riding lessons:

“It’s experience. You see things that are about to happen before they occur. I think there is a difference between being 19 and 59 years old. […] There is an indefinable feeling when you get a premonition that it is time to stop. It is a feeling.” (59 years, female, riding school).

The importance of communication and sharing information was mentioned by several employees from both riding schools and trotting stables. Communication about changes in routines and information about specific horses and incidents or accidents were considered important for the work environment and safety. However, communication and information spreading were commonly described as a challenge by the employees we interviewed. Some workplaces, mainly riding schools, were said to hold regular staff meetings where the work environment and safety were discussed, while most trotting stables relied on informal talks during coffee breaks or lunch. A general challenge was to ensure that all employees received the same information. Riding schools often have several part-time employees who work specific days and hours, which means that the employees are never all on-site at the same time, thus posing the risk of some workers missing out on information and decisions. One of the riding school workers reported:

“We [the staff] meet very seldomly. If it’s serious, I’ll send a message to the person starting work the next morning. In other situations, you talk to the manager. […] But it can fall through the cracks. A lot can happen when you are not there. You do not meet everyone every day. There can be a lack of information here.” (60 years, female, riding school). 

Several workplaces used Messenger or other smartphone communication applications to spread information, but some workers were critical of this as everyone then needs to have their phone with them all the time. Even if they do, there is a risk of some of the staff missing out on the information and you can never be sure that the information has been received by everyone. Employees at the riding schools stressed the importance of receiving the right information at the right time, not just for their safety but also that of their riders. For example, if a horse threw off a rider during a lesson, all the riding instructors should know this when they have lessons of their own so that they can pay special attention to that horse and possibly prevent more incidents. Employees at trotting stables also talked about the effects of missing information about changes in routines, which could negatively affect horse welfare.

#### 3.3.4. Trust in the Efficacy of Safety Systems (Dim7)

A few of the participating riding schools had a workplace safety representative, but only one employee reported that their workplace had a yearly safety evaluation. Thus, while the NOSACQ-50 mean score for Dim7 was relatively high for riding schools (3.62), there were indications in the interviews of shortcomings in the systematic work environment management. Risk assessments were commonly performed at the riding schools, but not always in a formalised way with the involvement of relevant staff and sometimes without regular follow-up. Risk awareness was generally described as high and many of the employees at riding schools reported that safety was a natural part of their everyday work and that they worked to prevent safety issues in the workplace:

“We are an association that really wants to engage with the working environment. Everyone is so willing and committed to working environment management. As soon as we have a new activity, we do a risk assessment. This is a dangerous job so it is important to keep to the existing rules. They are there for a reason, which is so that nothing happens. We are good at this, but we could do better and think even more safety. Near-misses are definitely something we talk about afterward, about whether could we think differently and do some things differently.” (30 years, female, riding school).

Employees at trotting stables reported they did not have a safety representative and did not do a yearly safety evaluation. A few of the employees at trotting stables said that they never or very seldom talk about safety and work environment issues with the manager or colleagues. None of the employees interviewed reported that they carry out risk assessments in the workplace, and safety issues were said to be discussed only if there had been an incident, as illustrated by the following statement:

“I know that they [the management] have a file with some form you have to fill in in the event of an accident, but it passes through your hands and then it’s forgotten. When something almost happens, we do not communicate at all, only if someone was injured.” (28 years, female, trotting stable).

None of the employees at either riding schools or trotting stables mentioned that they had any clear-cut goals for safety.

#### 3.3.5. Horsemanship and Safety

Horsemanship was an additional theme identified as linked to employees’ perceptions of factors affecting safety in the workplace, but not directly included in the seven dimensions in the NOSACQ-50.

As handling horses was perceived to be the most hazardous activity at both riding schools and trotting stables, employees’ skills were seen as an essential factor to safety. Many workers talked about the ability to read, sense and understand the horse, which is here called horsemanship. A worker at a trotting stable reported:

”I think about safety all the time. Have learned, over the years, a form of trial and error. The most important thing is to be able to read and sense the horse. If you don’t have that feeling, knowledge or experience, you can’t work in this job. It’s not possible to gain this knowledge from a book; it’s something you have to experience.” (33 years, female). 

This skill allows the handler to better predict the horse’s behaviour and stay one step ahead, which means being better prepared for the horse’s reactions to a given situation. It also includes understanding how to handle and care for the horse based on that horse’s character and needs, e.g., avoiding situations that may cause stress or arousal. Routines are also in place to support this, such as never leaving one horse alone in the paddock. However, the employees pointed out that you cannot control all situations and that accidents often happen due to circumstances beyond the staff’s control, for example, if a plastic bag suddenly startles a horse being led to pasture. This was also one reason why the employees believed that not all accidents can be prevented. They reported that they handle this unpredictability by striving to minimise the consequences of an incident, e.g., by wearing personal protective equipment like a helmet, gloves and safety boots.

## 4. Discussion

### 4.1. Study Outcomes

This study explored the safety climate perceptions of employees at Swedish riding schools and trotting stables using an explanatory sequential mixed-methods design that combined quantitative and qualitative methods. To date, the field of research on the safety climate and safety culture in the equine sector has been very limited. This study was intended as a first step toward addressing these issues and offering insights into the perceptions of employees at riding schools and trotting stables regarding risks, safety and safety management in their workplace. The results can be used to target areas in need of improvement and design effective interventions to improve the safety culture in the equine sector.

The responses to the NOSACQ-50 questionnaire indicated that the perceived safety climate at riding schools and trotting stables was generally good. For riding schools, the mean scores indicated a need for some improvement in only one dimension, workers’ safety prioritisation and risk non-acceptance. For trotting stables, the mean scores indicated a need for improvement in that same dimension and also in two other dimensions, the management’s safety prioritisation, commitment and competence and employees’ trust in the efficacy of safety systems. The scores given for the safety climate in this study were generally high in comparison with those reported in studies of other sectors, e.g. construction [51], agriculture [26], the production industry [52] and metal industry [53], and also with values in the NOSACQ-50 database covering 37 industrial sectors (Table 5). However, the equine sector still has a relatively high frequency of occupational injuries and is perceived as a high-risk work environment. The link between the workplace safety climate and safety-related performance and attitudes and injuries has been widely confirmed in previous studies [39,40,41,42] so our results were somewhat unexpected. Using a tool like the NOSACQ-50 can be a good way to assess the safety climate in a workplace, but it is important to keep in mind that it does not provide comprehensive information on the safety status of the workplace. Other factors, not covered in the NOSACQ-50, also affect safety, including e.g. the quality of the physical work environment, which is known to be somewhat neglected in the equine sector. Furthermore, the NOSACQ-50 is adapted to work across sectors and businesses, which means that sector- or business-specific aspects are not covered. When working with horses, the horse itself also plays a major role in the working environment and can be considered the main risk factor. The responses in interviews with employees, where horsemanship was identified as a separate theme that could not be related to any of the safety dimensions in the NOSACQ-50, supported this reasoning and highlighted the importance of a broader approach than merely considering the safety climate when assessing safety. 

In the NOSACQ-50 results, the safety climate at riding schools was scored higher than that at trotting stables, which is unsurprising when considering the differences between these contexts. The riding school is an environment where children, beginners and inexperienced riders learn about horses and horse riding, and safely encounter horses. This means that safety thinking is a natural part of the organisation, both in terms of the facilities and the use of suitable horses and ponies, even though risk assessments may not always be formalised and documented in the way required by the Swedish legislation. Swedish regulations on systematic work environment management require employers to examine the work environment, investigate ill-health and accidents, assess hazards, write an action plan, remedy the risks and monitor the effects of preventive measures [23]. In a trotting stable, the staff is usually experienced and works as a team when caring for the horses, without many visitors. The horses are competition horses that are often young and not always easily handled, and thus not suitable for beginners. This means that workplace safety management is mainly for the employees themselves, which may mean that safety is not prioritised to the same extent in trotting stables as in riding schools, which need to consider pupil safety. The results from the interviews reflected this, with employees at riding schools talking about safety as a self-evident part of their everyday work and reporting that they also felt a responsibility to teach safe practices to their pupils. 

The lower safety prioritisation in trotting stables was also evident in the results from the SWEM questionnaire completed by managers at the workplaces. The managers at trotting stables did not have a systematic work environment management system in place, and of particular note, made no effort to proactively investigate the working conditions or assess the risks of ill-health and accidents at work.

Safety awareness was high among employees at both riding schools and trotting stables, and handling horses was perceived as the most hazardous activity. There was a general acceptance of danger and imminent injury when interacting with horses and also a belief that the risk of injury was unavoidable. This is in line with Chapman et al. [18], who found that respondents (equestrians, i.e., not only professionals) de-emphasised the importance of safety-first principles during horse-related interactions. The interviews in the present study revealed a degree of risk normalisation, as some respondents viewed minor injuries as part of their work and even downplayed the severity of injuries they had experienced at work. The main explanation given for perceived difficulty in preventing injuries was that any interaction with a horse involves a certain risk, as horses are unpredictable and powerful animals. This reasoning by employees seemed to be the main underlying explanation for the lower safety climate score for the NOSACQ-50 dimension related to workers’ safety prioritisation and risk non-acceptance, especially among employees in trotting stables. A few employees also reported prioritising the health and safety of the horse over their own, as previously reported for riders engaged in high-risk equestrian sports [54]. The unpredictability and hazards of working with large animals, as recognised previously in the scientific literature [10], do not mean that risk-mitigation processes are ineffective since more recent literature has identified proactive elements that support safer horse handling [18]. Hawson et al. [55] suggested that the unpredictability of horses can be reduced by providing training in horse behaviour for horse riders and handlers, highlighting the role of learning theory.

Knowledge and experience were mentioned by many of the employees interviewed as relevant factors for safety in the workplace. In particular, horsemanship (i.e., the ability to read, communicate and interact with the horse) was viewed as essential for safety, as it can enable the handler to foresee certain behaviours in a given situation and train the horse in the correct way to ease the handling. This finding is in line with arguments put forward by Thompson et al. [9], who identified good horse-handling skills in combination with the use of personal protective equipment as key strategies for improving safety during horse-human interactions. Matching the horse to the handler (trotting stables) and choosing horses and ponies with a suitable temperament for inexperienced riders (riding schools) were also considered relevant by the employees interviewed in the present study. Matching the horse to the rider has gained some attention in the scientific literature, where e.g., temperament, behavioural traits and reactivity have proven to be important attributes of the horse [56,57,58]. Education and training are naturally one way of improving horsemanship, as suggested by Hawson et al. [55], but some employees interviewed here believed that these abilities cannot be learned from a book and must be acquired through “trial and error”. This process entails making mistakes and experiencing incidents before safe horse-handling skills are developed. It would be interesting to test these beliefs, but as also stressed by Chapman et al. [18], more research is needed to identify effective types of training, how best to encourage workers in the equestrian sector to implement safety principles and how to reach all members of the diverse group of equine professionals. In light of these challenges to communicating safety information within various equine cultures, Thompson et al. [9] pointed out a need to understand what motivates the behaviour of different equestrians and identify useful motivators for adopting protective behaviours, and in the long-term, improved safety values.

For trotting stables, the management’s safety prioritisation, commitment and competence were shown to represent an area in clear need of improvement. The SWEM questionnaire for managers confirmed these findings by indicating deficiencies in routines for proactive preventive assessment of risks in the work environment and reactive investigation of the causes of accidents at work, reflecting a lack of prioritisation of safety issues by managers. The riding schools generally worked more systematically with risk assessment and accident investigations, but there was room for improvements regarding e.g. involvement of employees and communication. Factors related to the management that were mentioned by the employees we interviewed were: orderliness, time pressure and time schedules, participation, communication and information. The importance of management and leadership for the workplace safety climate has been demonstrated in several previous studies [31,32]. In a meta-analysis of specific safety climate dimensions, Beus et al. [59] found the perceived management commitment to safety to be the most robust predictor of occupational injuries. Thus, management behaviour is as important as employee behaviour for a safety culture to be reliable, and the visible commitment of managers to safety can make a major difference to the quality of the employees’ work [29,60]. This may be especially important for trotting stables as the managers are usually present during the daily work, and therefore, have the opportunity to lead by example and set standards for safety. Hofmann and Stetzer [39] showed that work pressure, with-in group communication and the safety climate (management commitment to safety and workers’ involvement in safety activities), can be associated with unsafe behaviour, which supports the findings of interviews in the present study. Overall, the results show that efforts are needed to improve the safety information and manager training in trotting stables. It could be fruitful to develop methods and tools adapted to the equine sector that can aid managers in systematic work environment management, especially if these methods and tools also focus on improving employees’ safety motivation and participation. A participatory environment where employees are involved and encouraged to be creative and suggest innovative solutions is key [61]. Organisation of the work in terms of its order, resource management and communication would be interesting areas to target in intervention studies.

### 4.2. Limitations

When interpreting the results obtained in this study, some limitations should be kept in mind. A purposive sampling methodology was applied to collect data from a sample of riding schools and trotting stables, but the sample size was small compared to that in other survey studies that have used the NOSACQ-50 [26,51,52,53]. However, the present study was unique regarding its use of a mixed-methods approach and combination of quantitative and qualitative data. Due to the small sample size and the potential for selection bias, the results may not be generalisable; a larger sample of respondents could allow for a more accurate understanding of the safety climate in the equine sector. The sample represented a range of ages and work experiences, and workplaces of various sizes, which is a strength, but a majority of the respondents were female, which may have caused gender bias. However, the skewed gender distribution of the sample accurately reflects the equestrian workforce. Another consideration when interpreting the results is that the sampled workplaces may have agreed to participate in the study because they already have an interest in the work environment and safety issues, and thus may represent greater safety awareness than the average.

There was an imbalance in the number of interviews conducted with employees at riding schools and trotting stables. The smaller number of interviews at trotting stables may mean that we missed some information. However, informal conversations with the employees at workplaces where interviews were not conducted revealed no new information. Thus, we propose that saturation was reached, and additional interviews were not expected to add any new insights [48]. Furthermore, the interviews aimed to provide context and understanding to supplement the survey results, and so this uneven distribution is unlikely to have affected the results.

Triangulation [43], i.e., a combination of multiple methods and data, was used to gain a more complete understanding of the safety climate in riding schools and trotting stables. Although limited, the findings were compared with earlier research in the field, both for verification and to identify discrepancies, which improved the overall credibility of the results. Despite the descriptive nature of the results, they can be used to identify specific areas in need of improvement to enhance the safety climate in the equine sector.

## 5. Conclusions

In summary, the safety climate in the equine sector was generally found to be good, especially at riding schools, and employees at riding schools and trotting stables were well-aware of the risks related to their work. This shows that it is possible to have a good safety climate in a high-risk work environment. The riding schools commonly had routines for risk assessment and work environment management, though not in the formalised and systematic way that the Swedish regulations require. At trotting stables, such routines were often lacking, indicating a deficient prioritisation of safety by the management. The main area we identified as in need of improvement in the safety climate was the workers’ prioritisation and risk non-acceptance. The employees interviewed reported that they strive to work safely, but admitted taking calculated risks and sacrificing safety to gain time back or save their energy. There was a general acceptance of minor injuries as part of the job and a perception that many injuries cannot be prevented. This normalisation meant that incidents and accidents were ignored and thus not reported, discussed or followed up unless they were severe. This aspect of the equine safety culture should be targeted to improve safety and decrease occupational injuries. The management should foster a culture of safety awareness at all levels of the organisation and ensure that safety is an integral part of the daily work, not targeted solely when injuries occur. All injuries must be taken seriously and reported, and accident investigations should be used systematically to learn from negative events, identify factors contributing to occupational injuries and develop strategies for injury prevention. Employee participation and encouragement are further important factors. In these ways, through continuous improvements to the safety climate, the safety culture in the equine sector can be improved over time.

## Figures and Tables

**Table 1:** Summary of the data collection methods and sample size.

Information	Part 1	Part 2	Part 3
Type of data	Quantitative	Quantitative	Qualitative
Data collection	NOSACQ-50 ^1^ questionnaire	SWEM ^2^ questionnaire	Semi-structured interviews
Perspective	Employee	Employer	Employee
Sample (total *N*)	*N* = 66	*N* = 12	*N* = 47
Methods of analysis	Descriptive statistics and t-test	Descriptive statistics	Qualitative content analysis

^1^ Nordic Safety Climate Questionnaire. ^2^ Systematic Work Environment Management.

**Table 2:** Demographic characteristics of respondents to the Nordic Safety Climate Questionnaire (NOSACQ-50), systematic work environment management (SWEM) questionnaire and interviewees.

Information	Type	NOSACQ-50 (*N* = 66)	SWEM (*N* = 11) ^1^	Interviews (*N* = 47)
Age (years)	Mean	39	38	38
	Max.	65	58	60
	Min.	20	28	20
Gender (no. of respondents)	Female	51 (77%)	6 (55%)	40 (85%)
	Male	15 (23%)	5 (45%)	7 (15%)
Work experience (years)	Mean	9 ^2^	15	12
	Max.	41	30	40
	Min.	1	6	1
Type of organisation (no. of respondents)	Riding school	36 (55%)	6 (55%)	30 (64%)
	Trotting stable	30 (45%)	5 (45%)	17 (36%)

^1^ One missing datum. ^2^ Two missing data (*N* = 64) not shown.

**Table 3:** Sets of statements provided for each of the seven dimensions (Dim1–Dim7) in the Nordic Safety Climate Questionnaire (NOSACQ-50).

Dimensions	Description of the Dimension	Numbers and Content of Statements Provided
Dim1	Management safety prioritisation, commitment and competence	Nine statements to evaluate workers’ perceptions of safety management—e.g., management places safety before production.
Dim2	Management safety empowerment	Seven statements to evaluate workers’ perceptions of management empowerment and support to participate in overcoming safety issues—e.g., management involves employees in decisions regarding safety.
Dim3	Management safety justice	Six statements designed to estimate how workers perceive accidents’ management—e.g., management listens carefully to everyone who has been involved in an accident.
Dim4	Workers’ safety commitment	Six statements to indicate how workers perceive their own commitment to safety—e.g., we who work here try hard together to uphold a high level of safety.
Dim5	Workers’ safety prioritisation and risk non-acceptance	Seven statements indicating the workers’ risk-taking attitudes and safety prioritisation in their working tasks—e.g., we who work here never accept risk-taking even if the work schedule is tight.
Dim6	Safety communication, learning and trust in co-workers’ safety competence	Eight statements investigating how workers perceive the exchange of safety knowledge and experiences among themselves—e.g., we who work here learn from our experiences, to prevent accidents.
Dim7	Trust in the efficacy of safety systems	Seven statements to analyse workers’ perceptions of the benefits derived from safety planning, training, monitoring, etc.—e.g., we who work here consider it important to have clear-cut goals for safety.

**Table 4:** Criteria used to interpret the results of the Nordic Safety Climate Questionnaire (NOSACQ-50) as suggested by the National Research Centre for the Working Environment of Denmark [46].

Score	Level	Meaning
>3.30	Good	Maintaining and continuing developments of the safety climate dimension
3.00–3.30	Fairly good	The safety climate dimension is in slight need of improvement
2.70–2.99	Fairy low	The safety climate dimension is in need of improvement
<2.70	Low	The safety climate dimension is in great need of improvement

**Table 5:** Safety climate dimension (Dim) mean scores for all respondents (*N* = 66) and employees at riding schools (*N* = 36) and trotting stables (*N* = 30) separately (for a description of the dimensions, see Table 3). The column on the right presents data from the Nordic Safety Climate Questionnaire (NOSACQ-50) database covering 62,133 workers from 558 worksites and 37 industrial sectors internationally [46].

Dimension	Total	Ridings Schools	Trotting Stables	NOSACQ-50 Database
Dim1	3.38	3.46	3.27	3.06
Dim2	3.45	3.50	3.38	2.96
Dim3	3.50	3.50	3.49	2.99
Dim4	3.57	3.58	3.55	3.18
Dim5	2.93	3.08	2.76	2.98
Dim6	3.49	3.58	3.39	3.15
Dim7	3.41	3.62	3.16	3.23

**Table 6:** Differences in mean scores for the seven safety climate dimensions (Dim1–Dim7) in the Nordic Safety Climate Questionnaire (NOSACQ-50) between riding schools and trotting stables, females and males, two age groups and two work experience groups. Results of t-test analyses, where *t* indicates the t-test output and *p* indicates the level of significance.

Riding Schools (Dataset 1)—Trotting Stables (Dataset 2)														
	Dim1		Dim2		Dim3		Dim4		Dim5		Dim6		Dim7	
Dataset	1	2	1	2	1	2	1	2	1	2	1	2	1	2
Sample size	36	30	36	30	36	28	36	30	36	30	36	30	36	30
Mean value	3.46	3.27	3.50	3.38	3.50	3.50	3.58	3.55	3.08	2.76	3.58	3.39	3.62	3.16
StdD	0.58	0.41	0.53	0.46	0.61	0.52	0.43	0.39	0.60	0.59	0.42	0.33	0.38	0.56
*t*	1.566		1.005		0.073		0.282		2.155		2.092		3.814	
*p*	0.122		0.319		0.942		0.778		0.035 *		0.040 *		0.000 **	
**Females (Dataset 1)—Males (Dataset 2)**														
	**Dim1**		**Dim2**		**Dim3**		**Dim4**		**Dim5**		**Dim6**		**Dim7**	
Dataset	1	2	1	2	1	2	1	2	1	2	1	2	1	2
Sample size	51	15	51	15	50	14	51	15	51	15	51	15	51	15
Mean value	3.42	3.24	3.49	3.30	3.50	3.49	3.57	3.53	2.95	2.87	3.54	3.34	3.44	3.33
StdD	0.49	0.60	0.46	0.62	0.57	0.56	0.41	0.43	0.62	0.61	0.37	0.44	0.55	0.44
*t*	1.142		1.254		0.083		0.343		0.475		1.180		0.679	
*p*	0.258		0.214		0.934		0.733		0.637		0.075		0.500	
**20–35 Years Old (Dataset 1)—≥36 Years Old (Dataset 2)**														
	**Dim1**		**Dim2**		**Dim3**		**Dim4**		**Dim5**		**Dim6**		**Dim7**	
Dataset	1	2	1	2	1	2	1	2	1	2	1	2	1	2
Sample size	33	33	33	33	32	32	33	33	33	33	33	33	33	33
Mean value	3.30	3.46	3.43	3.47	3.50	3.50	3.60	3.53	2.86	3.01	3.46	3.53	3.30	3.53
StdD	0.53	0.49	0.50	0.51	0.52	0.61	0.35	0.46	0.65	0.58	0.38	0.40	0.57	0.46
*t*	−1.245		−0.289		−0.004		0.701		−1.018		−0.752		−1.824	
*p*	0.218		0.773		0.997		0.486		0.312		0.455		0.073	
**0–5 Years of Work Experience (Dataset 1)—≥6 Years of Work Experience (Dataset 2)**														
	**Dim1**		**Dim2**		**Dim3**		**Dim4**		**Dim5**		**Dim6**		**Dim7**	
Dataset	1	2	1	2	1	2	1	2	1	2	1	2	1	2
Sample size	39	25	39	25	37	25	39	25	39	25	39	25	39	25
Mean value	3.44	3.30	3.53	3.30	3.60	3.37	3.67	3.42	2.99	2.85	3.54	3.40	3.42	3.40
StdD	0.47	0.58	0.46	0.56	0.52	0.63	0.37	0.44	0.69	0.50	0.39	0.39	0.53	0.53
*t*	1.044		1.807		1.548		2.476		0.937		1.423		0.181	
*p*	0.300		0.076		0.127		0.016 *		0.352		0.160		0.857	

* *p* < 0.05; ** *p* < 0.001.

**Table 7:** Themes identified in qualitative analysis of data obtained in interviews with workers at riding schools and trotting stables, and the related safety dimension (Dim) in the Nordic Safety Climate Questionnaire (NOSACQ-50).

Theme	Safety Dimension	
Management	Dim1	Management safety prioritisation, commitment and competence
Risk awareness and acceptance	Dim5	Worker’s safety prioritisation and risk non-acceptance
Knowledge and experience	Dim6	Safety communication, learning and trust in co-workers’ safety competence
Communication and information	Dim6	Safety communication, learning and trust in co-workers’ safety competence
Safety routines	Dim7	Trust in the efficacy of safety systems
Horsemanship		No related safety dimension

## Data Availability

The data presented in this study are available on request from the corresponding authors. The data are not publicly available due to confidentiality concerns, as the General Data Protection Regulation (GDPR) requires researchers to protect the identity of participants.
